# Revealing the electronic, optical and photocatalytic properties of PN-M_2_CO_2_ (P = Al, Ga; M = Ti, Zr, Hf) heterostructures

**DOI:** 10.1039/d3na00017f

**Published:** 2023-02-02

**Authors:** M. Munawar, M. Idrees, Tahani A. Alrebdi, B. Amin

**Affiliations:** a Department of Physics, Abbottabad University of Science & Technology Abbottabad 22010 Pakistan binukhn@gmail.com; b Department of Physics, College of Science, Princess Nourah Bint Abdulrahman University P.O. Box 84428 Riyadh 11671 Saudi Arabia taalrebdi@pnu.edu.pk

## Abstract

Using DFT, the electronic structure, optical, and photocatalytic properties of PN (P = Ga, Al) and M_2_CO_2_ (M = Ti, Zr, Hf) monolayers and their PN-M_2_CO_2_ van der Waals heterostructures (vdWHs) are investigated. Optimized lattice parameters, bond length, bandgap, conduction and valence band edges show the potential of PN (P = Ga, Al) and M_2_CO_2_ (M = Ti, Zr, Hf) monolayers in photocatalytic applications, and the application of the present approach to combine these monolayers and form vdWHs for efficient electronic, optoelectronic and photocatalytic applications is shown. Based on the same hexagonal symmetry and experimentally achievable lattice mismatch of PN (P = Ga, Al) with M_2_CO_2_ (M = Ti, Zr, Hf) monolayers, we have fabricated PN-M_2_CO_2_ vdWHs. Binding energies, interlayer distance and AIMD calculations show the stability of PN-M_2_CO_2_ vdWHs and demonstrate that these materials can be easily fabricated experimentally. The calculated electronic band structures show that all the PN-M_2_CO_2_ vdWHs are indirect bandgap semiconductors. Type-II[-I] band alignment is obtained for GaN(AlN)-Ti_2_CO_2_[GaN(AlN)-Zr_2_CO_2_ and GaN(AlN)-Hf_2_CO_2_] vdWHs. PN-Ti_2_CO_2_ (PN-Zr_2_CO_2_) vdWHs with a PN(Zr_2_CO_2_) monolayer have greater potential than a Ti_2_CO_2_(PN) monolayer, indicating that charge is transfer from the Ti_2_CO_2_(PN) to PN(Zr_2_CO_2_) monolayer, while the potential drop separates charge carriers (electron and holes) at the interface. The work function and effective mass of the carriers of PN-M_2_CO_2_ vdWHs are also calculated and presented. A red (blue) shift is observed in the position of excitonic peaks from AlN to GaN in PN-Ti_2_CO_2_ and PN-Hf_2_CO_2_ (PN-Zr_2_CO_2_) vdWHs, while significant absorption for photon energies above 2 eV for AlN-Zr_2_CO_2_, GaN-Ti_2_CO_2_ and PN-Hf_2_CO_2_, give them good optical profiles. The calculated photocatalytic properties demonstrate that PN-M_2_CO_2_ (P = Al, Ga; M = Ti, Zr, Hf) vdWHs are the best candidates for photocatalytic water splitting.

## Abstract 2

Using DFT, the electronic structure, optical, and photocatalytic properties of PN (P = Ga, Al) and M_2_CO_2_ (M = Ti, Zr, Hf) monolayers and their PN-M_2_CO_2_ van der Waals heterostructures are investigated.

## Graphical abstract

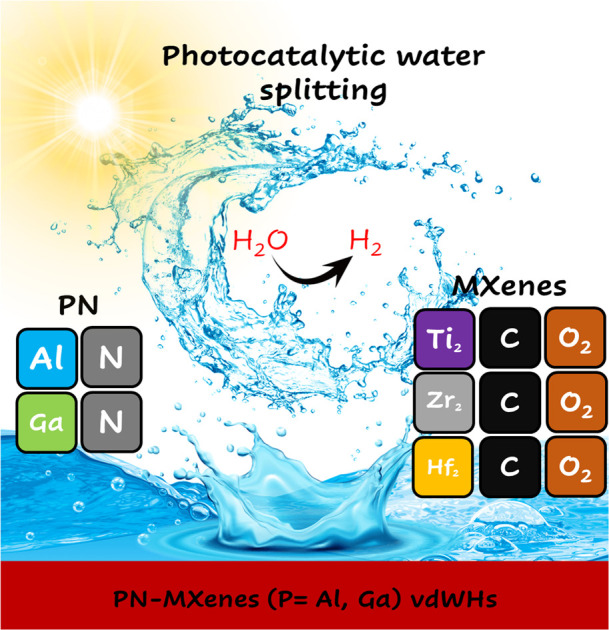

## I. Introduction

Photocatalytic water splitting is considered as the most suitable way to convert sunlight into chemical energy in the form of hydrogen.^1^ Therefore, understanding the mechanism of photocatalytic water splitting and exploring new photocatalysts are of interest for both fundamental research and industrial applications.^2^ Light harvesting, separation, and diffusion of photogenerated carriers (electrons and holes) to the surface, and hydrogen and oxygen evaluation reactions are the major steps in photocatalysis. A suitable bandgap^3^ to strengthen (weaken) electron–hole separation (recombination) time,^4^ and band edge positions (straddling the redox potential of water) of a potential semiconductor photocatalyst, are key in order to achieve the overall water splitting process.^5^

In the case of two-dimensional (2D) materials, a large surface area provides a shorter route for photogenerated electron–hole pairs to flow to the surface and facilitate a redox reaction, hence increasing the lifetime of carriers. Therefore, 2D materials are considered as pioneer candidates for photocatalytic water splitting as compared to their bulk counterparts.^6^ Performance of 2D materials is not limited to photocatalytic water splitting, and is also gaining much attention in both optoelectronics and thermoelectric device applications.^7,8^ In the family of 2D materials, MXenes produced by etching the A layer from the MAX phase,^9^ have a rich surface with functional groups (O, OH, F), responsible for excellent electronic,^10^ optical,^11^ electrochemical,^12^ and mechanical properties^13,14^ in catalysis,^15^ energy storage,^16^ batteries,^17^ photocatalytic water splitting^18^ and other related fields.^19^ Another group of the 2D family, PN (P = Al, Ga) monolayers, are excellent candidates as compared to their bulk counterparts, due to their thin structure, high active surface, high stability, large energy bandgap, blue-shifted photoluminescence peaks, high internal quantum efficiency and transfer of charge from Al/Ga to N.^20–23^ Experimentally, AlN is prepared by CVD, MBE and PVT techniques,^24^ while GaN is prepared by a migration enhanced encapsulated growth technique.^25^ Structural stability of the PN (P = Al, Ga) monolayer are further confirmed by phonon spectrum calculations.^26,27^ Strain engineering can also be performed to tune the bandgap of PN monolayers without structure distortion.^28,29^ PN (P = Al, Ga) nanowire,^30,31^ nanosheets^32,33^ and nanoribbons,^34,35^ have also been reported with fascinating properties.

The emergence of vdWHs in the 2D field offers a new way to combine different monolayers for novel electronic, optoelectronic, and photocatalytic applications.^36,37^ Some vdWHs based on MXene, AlN, and GaN like MoS_2_/MXene,^38^ TiO_2_/Mxenes,^39^ MXene/graphene,^40^ TMDCs/MXenes,^41^ SiS/MXenes,^42^ BSe/MXenes,^43^ AlGaN/GaN,^44^ MoSe_2_/GaN,^45^ AlN/GaN,^46^ WS_2_/GaN,^47^ ZnO/GaN,^48^ graphene/AlN,^49^ BP/AlN,^50^ and AlN/InSe,^51^ have already been fabricated for practical device applications. Electronic structure, optical properties and overall water splitting performance of GaN/Hf_2_CO_2_ and AlN/Hf_2_CO_2_ vdWHs have been investigated in ref. 52 and 53. A type-II band alignment for full water splitting is also observed in boron phosphide-blue phosphorene,^54^ C_2_N-based type-II heterojunctions^55^ and other 2D materials.^56^

From this aspect, surprisingly no investigation has addressed PN-M_2_CO_2_ (P = Al, Ga; M = Ti, Zr) vdWHs. Therefore, based on the same hexagonal symmetry, small lattice mismatch and unique performance of all MXenes of group M_2_CO_2_ (M = Ti, Zr, Hf) and PN (P = Al, Ga) monolayers, we combine these monolayers, in the form PN-M_2_CO_2_ (P = Al, Ga; M = Ti, Zr, Hf) vdWHs. A detailed study is gained to explore the electronic structure, optical and photocatalytic properties of PN-M_2_CO_2_ (P = Al, Ga; M = Ti, Zr, Hf) vdWHs. Our investigation revealed that all these six heterostructures are promising candidates for photocatalytic and optoelectronic devices.

## II. Computational details

We employ DFT ^57^ in VASP,^58^ with PBE ^59^ and HSE06 ^60^ functionals for both atomic relaxation and electronic bandstructure calculations. These calculations are performed with a conjugate gradient algorithm to minimize the atomic forces (energy) with a tolerance of 0.01 eV Å^−1^ (10^−4^ eV). A cut-off energy of 500 eV, *Γ*-centered *k*-point mesh of 8 × 8 × 1, and a vacuum of 25 Å along the *z*-direction were used to ensure a negligible interaction between adjacent layers.

AIMD ^61^ simulations were used to investigate the thermal stabilities of PN-M_2_CO_2_ (P = Al, Ga; M = Ti, Zr, Hf) vdWHs through the nose thermostat algorithm at 300 K. The system was allowed to relax for 12 picoseconds with a time step of 3 femtoseconds.^62,63^

The Bethe–Salpeter equation (BSE) is also solved in the GW_0_ calculation^64^ using the epsilon package^65^ in Quantum Espresso ^66^ to obtain *ε*_2_(*ω*). Furthermore, using the turbo Lanczos algorithm,^67^ we investigated the absorption coefficient (*α*) to understand the optical behaviour of these systems in detail.^68^

## III. Results and discussion

**Fig. 1:**
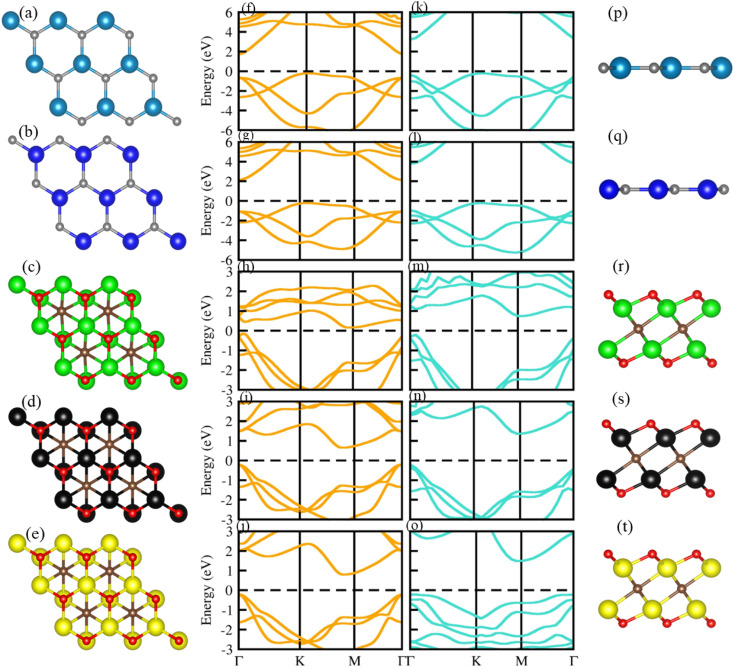
Geometrical structure (top view in first column), electronic bandstructure (PBE in second column; HSE06 in third column) and side view (fourth column) of GaN ((a), (f), (k), (p)), AlN ((b), (g), (i), (q)), Ti_2_CO_2_ ((c), (h), (m), (r)), Zr_2_CO_2_ ((d), (j), (n), (s)) and Hf_2_CO_2_ ((e), (j), (o), (t)).

**Fig. 2:**
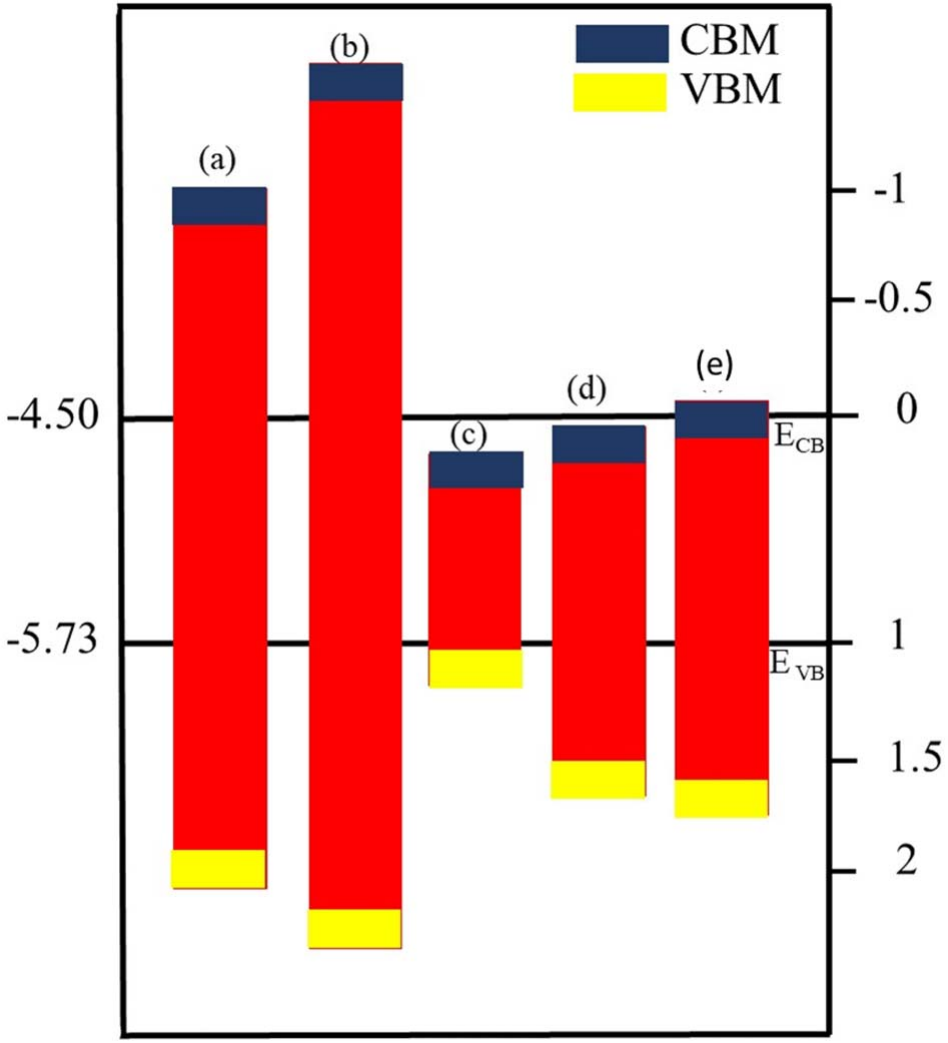
Band alignment for the valence band (VB) and conduction band (CB) edge of (a) GaN, (b) AlN, (c) Ti_2_CO_2_, (d) Zr_2_CO_2_ and (e) Hf_2_CO_2_ monolayers.

Optimized geometry (top and side view) and electronic bandstructure in Fig. 1, show that PN (P = Al, N) and M_2_CO_2_ (M = Ti, Zr, Hf) monolayers are indirect bandgap semiconductors. In the case of the PN (P = Ga, Al) monolayer, the CBM (VBM) lies at the *Γ*(*K*)-point, while for M_2_CO_2_ the VBM lies at the *Γ*-point and the CBM is between the *K* and *M* point of the first BZ, consistent with ref. 43 and 69. The photocatalytic response in Fig. 2, shows that Ti_2_CO_2_ and Zr_2_CO_2_ cross (fail to cross) the valence (conduction) band edge, while both the PN and Hf_2_CO_2_ monolayers cross both the conduction and valence band edges (see also Table 1), in agreement with ref. 43. These results show the potential of PN (M = Ga, Al) and M_2_CO_2_ (M = Ti, Zr, Hf) monolayers in photocatalytic applications and the authenticity of the present approach to combine these monolayers to form vdWHs for efficient electronic, optoelectronic and photocatalytic applications.

**Table 1:** Lattice constant (*a* in Å), bond length (Ga–N, Al–N, M–O and M–C in Å), band gap (*E*_g_ in eV), and conduction and valence band edge potentials (*E*_CB_ and *E*_VB_ in eV) for PN (P = Ga, Al) and M_2_CO_2_ (M = Ti, Zr, Hf) monolayers

Monolayers	GaN	AlN	Ti_2_CO_2_	Zr_2_CO_2_	Hf_2_CO_2_
*a*	3.255	3.288	3.01	3.31	3.27
Ga–N	1.894	—	—	—	—
Al–N	—	1.807	—	—	—
M–O	—	—	1.97	2.119	2.091
M–C	—	—	2.21	2.359	2.332
*E*_g_ (PBE)	1.98	2.35	0.3	0.865	0.99
*E*_g_ (HSE06)	3.23	3.98	0.95	1.57	1.69
*E*_CB_	−1.0022	−1.6823	0.3398	0.0798	−0.002
*E*_VB_	2.1977	2.3176	1.2898	1.6498	1.6923

**Fig. 3:**
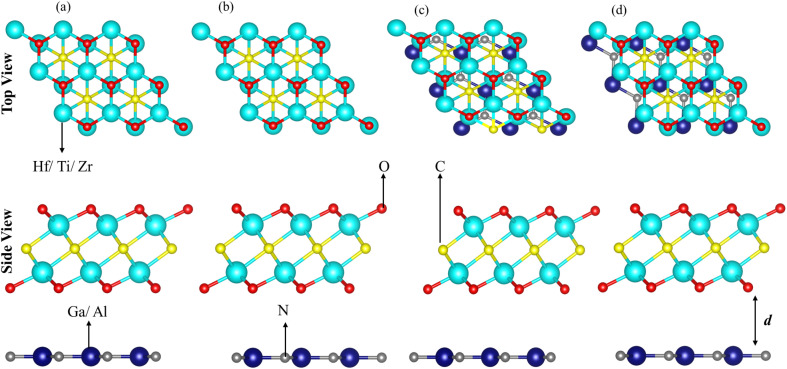
Possible stacking configurations of PN (P = Ga, Al) and M_2_CO_2_ (M = Ti, Zr, Hf) vdWHs.

Although the lattice mismatch of GaN(AlN) with Ti_2_CO_2_ is 7.52 (8.45)%, with Zr_2_CO_2_ is 1.66 (0.90)% and with Hf_2_CO_2_ is 0.45 (0.30)%, these are experimentally achievable^70^ in the fabrication of PN-M_2_CO_2_ vdWHs. But controlling the orientation of layers while fabricating vdWHs using mechanical exfoliation is quite difficult. Moreover, the electronic bandstructure is very sensitive to the stacking of layers. Therefore, we fabricated four different stacking patterns of PN-M_2_CO_2_ (P = Ga, Al; M = Ti, Zr, Hf) vdWHs, as presented in Fig. 3. In stacking (a), the M(O) atom of the M_2_CO_2_ layer is placed on the top of Ga/Al(N) atom of the PN layer; in stacking (b), the M(O) atom of the M_2_CO_2_ layer is placed on top of the N(Ga/Al) atom of the PN layer; in stacking (c), the M(C) atom of the M_2_CO_2_ layer is placed on top of the N(Ga/Al) of the PN layer; and stacking (d) is the reciprocal of stacking (c). The magnitude of the binding energy (*E*_b_ = *E*_PN-M_2_CO_2__ − *E*_M_2_CO_2__ − *E*_PN_, where *E*_PN-M_2_CO_2__ is the total energy of the vdWHs, *E*_M_2_CO_2__ is the total energy of the isolated M_2_CO_2_ monolayer, and *E*_PN_ is the total energy of the isolated PN monolayer) and the interlayer distance, show the disparity between different stacking patterns. The shorter the interlayer distance and the smaller the binding energy, corresponds to a more stable configuration, see Table 2. Therefore, stacking (d) of PN-M_2_CO_2_ vdWHs is the most favorable stacking configuration. Negative binding energy confirms that formation of PN-M_2_CO_2_ vdWHs are exothermic.^71–73^ The optimized lattice constants and bond lengths for the most stable stacking configuration are given in Table 3.

**Table 2:** Binding energy (*E*_b_ in eV) and interlayer distance (*d* in Å) of the PN (P = Ga, Al)-M_2_CO_2_ (M = Ti, Zr, Hf) vdWHs in different stacking configurations

Stacking	GaN-Ti_2_CO_2_	GaN-Zr_2_CO_2_	GaN-H_2_CO_2_	AlN-Ti_2_CO_2_	AlN-Zr_2_CO_2_	AlN-Hf_2_CO_2_
*E*_b_(a)	−0.808	−0.116	−0.141	−0.739	−0.962	−0.064
*d*	3.26	3.33	3.37	4.305	3.28	3.289
*E*_b_(b)	−0.818	−0.157	−0.182	−0.324	−0.359	−0.064
*d*	3.24	3.246	3.24	3.31	3.3	3.39
*E*_b_(c)	−0.813	−0.117	−0.003	−0.055	−0.939	−0.064
*d*	3.27	3.28	3.29	3.28	3.25	3.29
*E*_b_(d)	−0.833	−0.217	−0.382	−0.884	−1.062	−0.104
*d*	3.05	3.21	3.19	3.24	3.22	3.23

**Table 3:** Lattice constant (*a* in Å), bandgap (*E*_g_ in eV), potential difference (Δ*V* in eV), work function (*ϕ* in eV), effective mass (
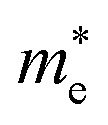
 and 
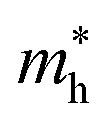
), conduction and valence band edges (*E*_CB_ and *E*_VB_ in eV) of PN-M_2_CO_2_ (P = Ga, Al; M = Ti, Zr, Hf) vdWHs

Heterostructure	*a*	*E*_g_-PBE	*E*_g_-HSE	Δ*V*	*ϕ*	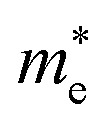	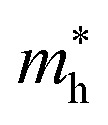	*E*_CB_	*E*_VB_
GaN-Ti_2_CO_2_	3.31	0.14	0.65	0.71	6.46	1.07	2.59	0.35	1.0
GaN-Zr_2_CO_2_	3.28	0.81	1.62	10.44	7.60	1.30	2.43	−0.11	1.51
GaN-Hf_2_CO_2_	3.26	0.91	1.69	2.44	5.67	1.20	1.40	−0.15	1.53
AlN-Ti_2_CO_2_	3.15	0.60	1.15	11.01	6.17	3.44	3.01	0.12	1.27
AlN-Zr_2_CO_2_	3.30	0.84	1.85	0.41	7.31	1.47	2.31	−0.03	1.46
AlN-Hf_2_CO_2_	3.28	0.88	1.79	8.02	5.50	1.24	1.54	−0.183	1.60

**Fig. 4:**
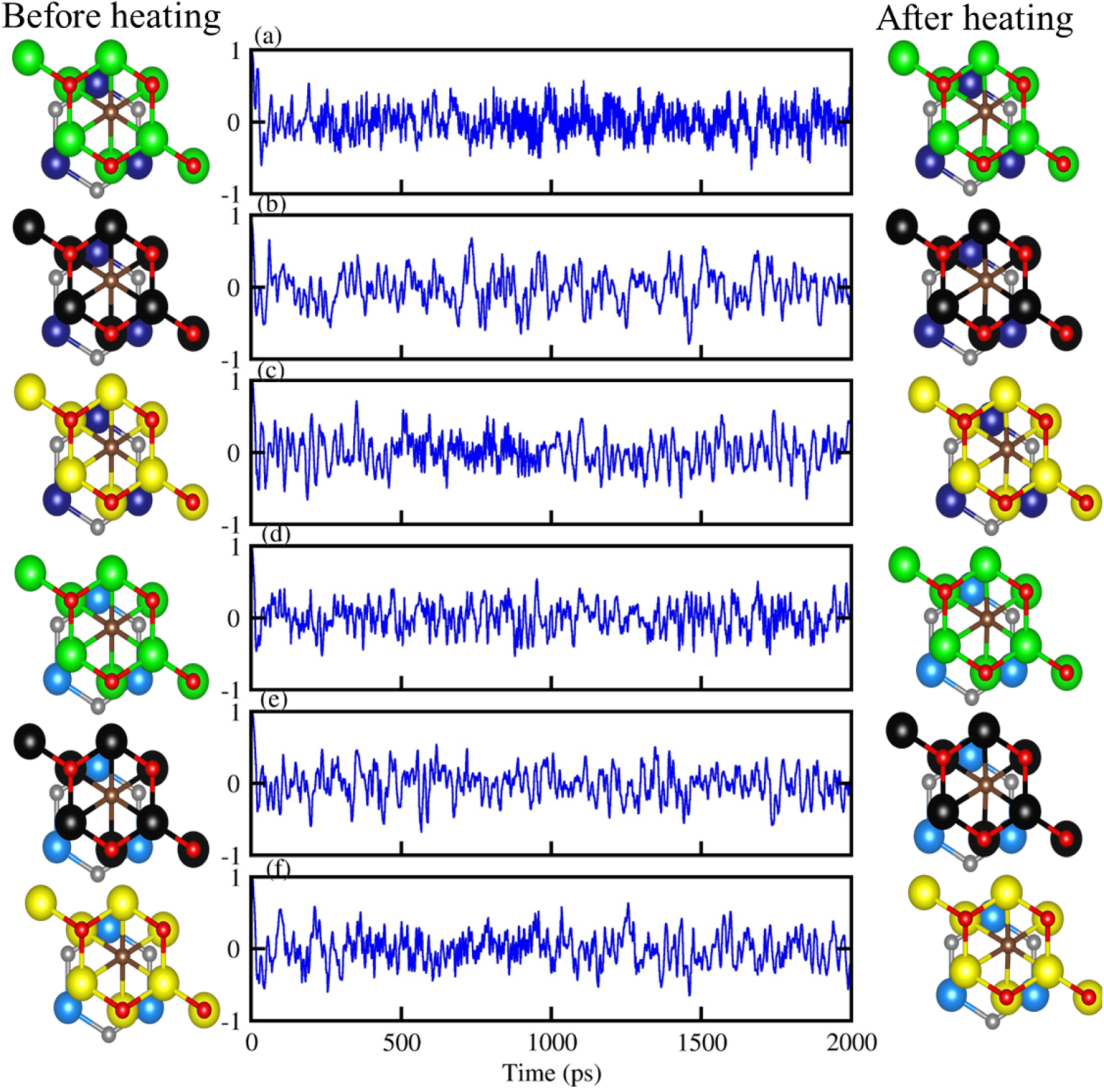
Geometrical structure before heating (first column), with fluctuating energy (second column) and after heating (third column) of: (a) GaN-Ti_2_CO_2_, (b) GaN-Zr_2_CO_2_, (c) GaN-Hf_2_CO_2_, (d) AlN-Ti_2_CO_2_, (e) AlN-Zr_2_CO_2_ and (f) AlN-Hf_2_CO_2_ vdWHs using AIMD simulation.

Further, the thermal stability of stacking (d) of PN-M_2_CO_2_ vdWHs, is verified using AMID simulation with 3 × 3 × 1 supercell, (see top view of the structures, before and after heating in Fig. 4). It is clear that after heating for 5 ps at 1 fs time stops at 300 K, all six of the most stable configuration patterns of PN-M_2_CO_2_ vdWHs show no broken bonds (Fig. 4, column 3), while free energy oscillates slightly (Fig. 4, column 2), and hence confirms the thermal stability of understudy vdWHs. Therefore, stacking (d) is the most stable stacking configuration and is further investigated in detail.

**Fig. 5:**
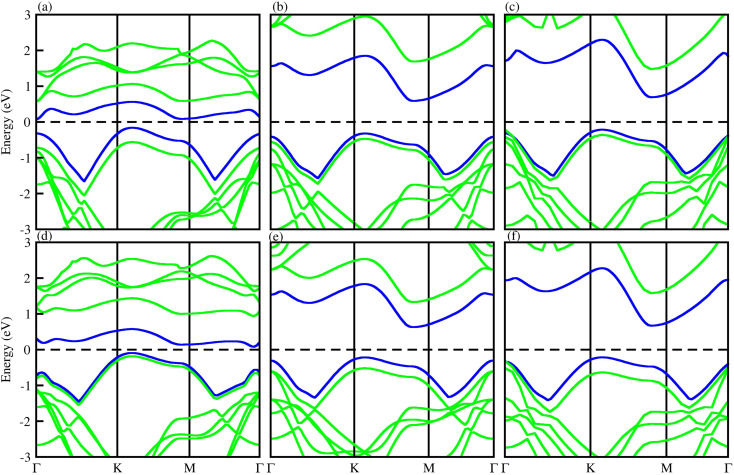
PBE (blue) and HSE06 (green) band structures of (a) GaN-Ti_2_CO_2_, (b) GaN-Zr_2_CO_2_, (c) GaN-Hf_2_CO_2_, (d) AlN-Ti_2_CO_2_, (e) AlN-Zr_2_CO_2_ and (f) AlN-Hf_2_CO_2_ vdWHs.

The behavior of the bandstructure and bandgap values are dependent on exchange correlation functionals.^74^ Therefore, we have calculated the bandstructure of PN-M_2_CO_2_ vdWHs using both PBE and HSE06 functionals, see Fig. 5, with the bandgap values listed in Table 3. One can see that HSE06 band gap values are larger than those of the PBE method, as the PBE approach underestimates the band gap values of semiconductors. For PN-Ti_2_CO_2_(GaN-Zr_2_CO_2_ and PN-Hf_2_CO_2_) vdWHs, the VBM lies at the *K*–*M*(*Γ*)-point and the CBM at the *Γ*(*K*–*M*)-point of the first BZ. For AlN-Zr_2_CO_2_, both the VBM and CBM lie at the *K*–*M* point of the first BZ. These results show that all the vdWHs are indirect bandgap semiconductors, where electrons must undergo a significant change in momentum for a photon of energy (*E*_g_) to create an electron–hole pair.^75^ In the case of indirect bandgap semiconductors, the electron interacts with the photon (phonon) to gain (gain or lose) energy (momentum). Hence, the position of the CBM and VBM can be aligned using suitable light according to the bandgap, and a transition is possible at lower energy using phonons, making them useful for laser applications.^76–78^

**Fig. 6:**
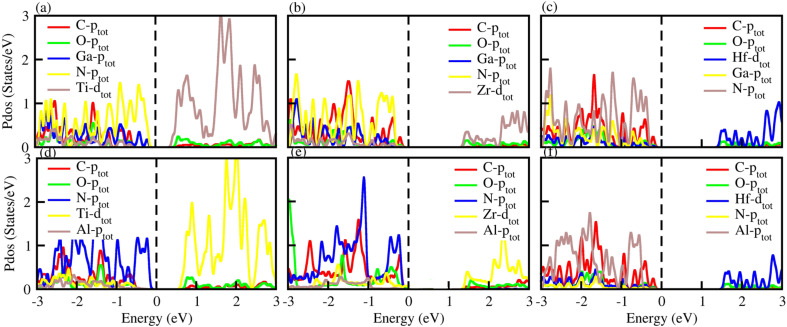
PDOS of (a) GaN-Ti_2_CO_2_, (b) GaN-Zr_2_CO_2_, (c) GaN-Hf_2_CO_2_, (d) AlN-Ti_2_CO_2_, (e) AlN-Zr_2_CO_2_ and (f) AlN-Hf_2_CO_2_ vdWHs.

To check the atomic states in the CBM/VBM at the Fermi level and band alignment, we plotted the partial density of states (PDOS) for PN-M_2_CO_2_ (P = Ga, Al; M = Hf, Ti and Zr) vdWHs, see Fig. 6. In the case of PN-Ti_2_CO_2_, the VBM (CBM) is from the N_P_ (Ti_d_) state of the PN(Ti_2_CO_2_) monolayer confirming type-II band alignment. The relative band position allows the mobility of electron (holes) from the conduction (valence) band of Ti_2_CO_2_(PN) to the conduction (valence) band of the PN(Ti_2_CO_2_) monolayer, offering a potential technique for charge separation. Hence, PN-Ti_2_CO_2_ vdWHs are promising candidates for solar energy conversion.^79^ In the case of PN-Zr_2_CO_2_(Hf_2_CO_2_), the VBM and CBM is due to the C_p_ state and the Zr/Hf_d_ state, indicating type-I band alignment, as the CBM and VBM is from the same monolayer. Both the electron and hole from the PN is transported to Zr_2_CO_2_(Hf_2_CO_2_), so would not provide good charge separation. However, since the Fermi level of semiconductors tune the band alignment (for non-intrinsic semiconductors), the carrier (electrons and holes) movement can be hindered in a certain direction (depending on the internal electric field direction); that is, electrons may be transported to Zr_2_CO_2_(Hf_2_CO_2_), but hole movement to Zr_2_CO_2_(Hf_2_CO_2_) is hindered. Hence, the resulting charge separation makes the materials the best candidates for light emission applications.^80^

**Fig. 7:**
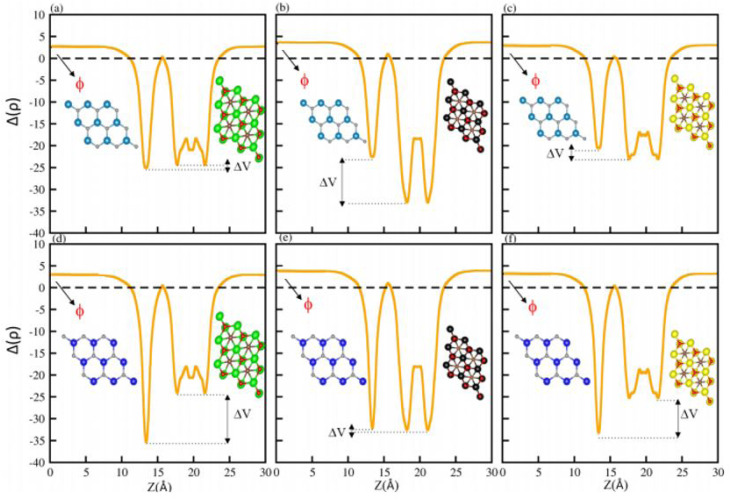
Average electrostatic potential of (a) GaN-Ti_2_CO_2_, (b) GaN-Zr_2_CO_2_, (c) GaN-Hf_2_CO_2_, (d) AlN-Ti_2_CO_2_, (e) AlN-Zr_2_CO_2_ and (f) AlN-Hf_2_CO_2_ vdWHs. The work function (*ϕ*) and potential drop (Δ*V*) are highlighted.

The average electrostatic potential shown in Fig. 7 shows that in the case of the PN-Ti_2_CO_2_ (PN-Zr_2_CO_2_) vdWH, the PN(Zr_2_CO_2_) monolayer has deeper potential than the Ti_2_CO_2_(PN) monolayer, indicating that charge is transferred from the Ti_2_CO_2_(PN) monolayer to the PN(Zr_2_CO_2_) monolayer. Meanwhile, in the case of GaN-Hf_2_CO_2_ (AlN-Hf_2_CO_2_), the Hf_2_CO_2_(AlN) monolayer has deeper potential than GaN(Hf_2_CO_2_), showing that charge is transferred from GaN(Hf_2_CO_2_) to Hf_2_CO_2_(AlN). The potential drop across the vdWHs (see Table 3) separates charge carriers (electron and holes) at the interface.^81,82^ Fabrication of the vdWHs enhances the electronic properties of the corresponding monolayers, hence effectively modulating the work function (*ϕ*). It also changes with strain and electric field engineering and doping.^83^ So, we have calculated the work function (*ϕ* = *E*_vec_ − *E*_F_, where *E*_vec_ (*E*_F_) represent the vacuum potential which is derived from the electrostatic potential (Fermi energy)) for all PN-M_2_CO_2_ (P = Ga, Al; M = Ti, Zr, Hf) vdWHs, see Fig. 7 and Table 3. The calculated work function of PN-M_2_CO_2_ vdWHs is almost the average of the corresponding monolayers (PN (P = Ga, Al) and M_2_CO_2_ (M = Ti, Zr, Hf)), which is efficient for the transfer of charge. The effective mass of the electron and holes for PN-M_2_CO_2_(P = Ga, Al; M = Ti, Zr, Hf) vdWHs are also calculated and are presented in Table 3. Materials with lower effective mass and high carrier mobility, are strongly preferred for high performance electronic devices,^84^ while a large effective mass greatly suppresses the quantum tunnelling, hence these materials are more appealing for logic devices.^85^ For AlN-Ti_2_CO_2_ the effective mass of electrons and holes is greater and is suggested for logic devices, while for the rest of the vdWHs the effective mass of electrons and holes is less than AlN-Ti_2_CO_2_, and is considered to be the best for high performance electronic devices.

**Fig. 8:**
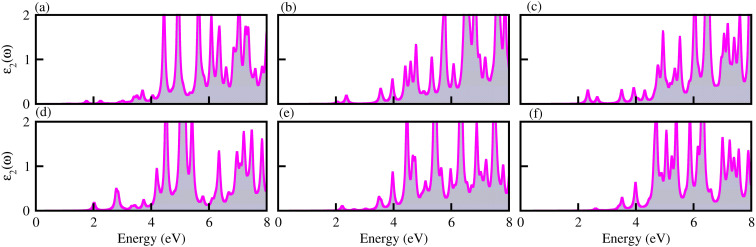
*ε*_2_(*ω*) of (a) GaN-Ti_2_CO_2_, (b) GaN-Zr_2_CO_2_, (c) GaN-Hf_2_CO_2_, (d) AlN-Ti_2_CO_2_, (e) AlN-Zr_2_CO_2_ and (f) AlN-Hf_2_CO_2_ vdWHs.

**Fig. 9:**
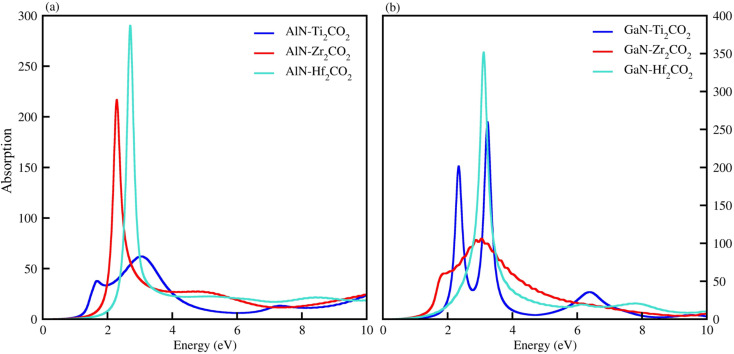
*α*(*ω*) of (a) AlN-Ti_2_CO_2_, AlN-Zr_2_CO_2_ and AlN-Hf_2_CO_2_ and (b) GaN-Ti_2_CO_2_, GaN-Zr_2_CO_2_, GaN-Hf_2_CO_2_, vdWHs.

The optical properties of materials define how materials react to incident electromagnetic radiation. We used DFT-PBE simulations to calculate optical features of PN-M_2_CO_2_ (P = Al, Ga; M = Ti, Zr, Hf) vdWHs such as the imaginary part of the dielectric function (*ε*_2_(*ω*) in Fig. 8) and absorption coefficients (*α*(*ω*) in Fig. 9). The observed excitonic peaks appear at 2.0 (1.9) eV and 2.8 (2.2) eV for AlN-Ti_2_CO_2_ (GaN-Ti_2_CO_2_) vdWHs; at 2.2 (2.2) eV and 3.7 (3.8) eV for AlN-Zr_2_CO_2_ (GaN-Zr_2_CO_2_) vdWHs; at 2.7 (2.2) eV and 3.7 (2.3) eV for AlN-Hf_2_CO_2_ (GaN-Hf_2_CO_2_) vdWHs. A red (blue) shift is observed in the position of excitonic peaks from AlN to GaN in PN-Ti_2_CO_2_ and PN-Hf_2_CO_2_ (PN-Zr_2_CO_2_) vdWHs. Furthermore, absorption coefficient *α* (μm^−1^) provides the photon power attenuation when it passes through the material. It also tells us how far light of specific energy and wavelength can spike the surface of the material before being absorbed.^86,87^ Although absorption is mainly determined by *ε*_2_(*ω*), this generalization is obviously not valid if the medium has very large *α*(*ω*).^88^ Therefore, we have further calculated the *α*(*ω*) of PN-M_2_CO_2_ (P = Ga, Al; M = Ti, Zr, Hf) vdWHs, as presented in Fig. 9, using the Lanczos program to solve the recursively quantum Liouville equation in the standard batch representation, which allows us to avoid interactions and multiplications of large matrices.^89^ The obtained values for *α*(*ω*) show that there is significant absorption for photon energies above 2 eV for AlN-Zr_2_CO_2_, GaN-Ti_2_CO_2_ and PN-Hf_2_CO_2_, making them candidates with a good optical profile. While in the case of AlN-Ti_2_CO_2_ and GaN-Zr_2_CO_2,_ the absorption is for photon energies above 1.8 eV. It is straight forward to understand the difference in spectra resulting from the bandstructure.^90^

**Fig. 10:**
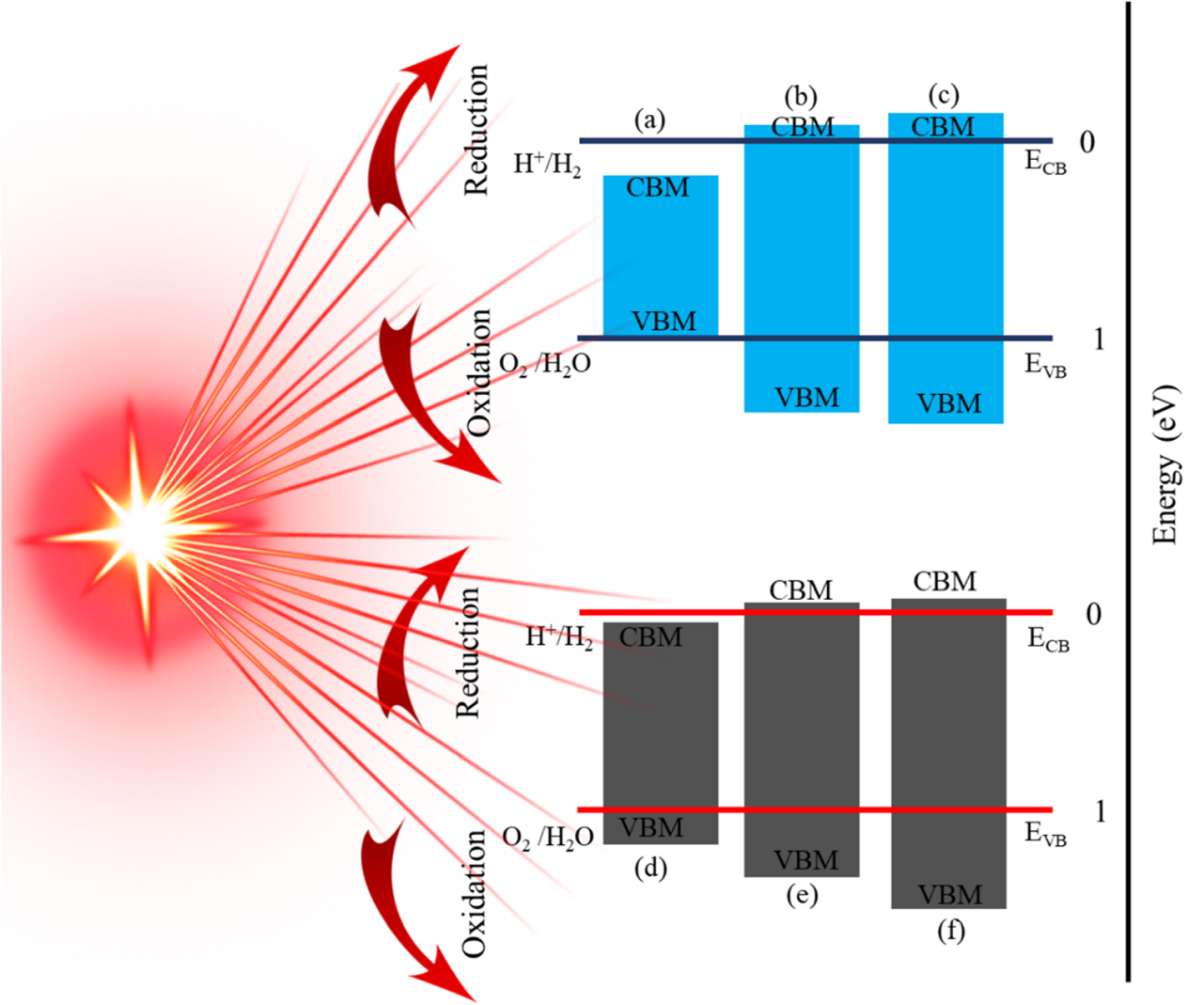
Band alignment for the valence band (VB) and conduction band (CB) edge of (a) GaN-Ti_2_CO_2_, (b) GaN-Zr_2_CO_2_, (c) GaN-Hf_2_CO_2_, (d) AlN-Ti_2_CO_2_, (e) AlN-Zr_2_CO_2_ and (f) AlN-Hf_2_CO_2_, vdWHs.

To utilize solar energy for environmentally friendly applications, it is necessary to design novel materials with visible light absorption. Therefore, to explore the ability of PN-M_2_CO_2_ (P = Al, Ga; M = Ti, Zr, Hf) vdWHs for water splitting, we calculated the photocatalytic properties by using Mullikan electronegativity.^91–93^ The conduction band (CB) and valence band (VB) edge position of the semiconductors are the two most important parameters to determine photocatalytic activity using under-stimulated sunlight irradiation.^94^ We denote it as H^+^/H_2_ for reduction and O_2_/H_2_O for oxidation, as shown in Fig. 10. If *E*_CB_ is positioned more negatively than the energy of the H_2_/^+^ potential and the *E*_VB_ is more positive than the energy of the O_2_/H_2_O potential, then water molecules can be easily split into H_2_ and O_2_.^95^ In GaN-Zr_2_CO_2_, GaN-Hf_2_CO_2_, AlN-Zr_2_CO_2_, and AlN-Hf_2_CO_2_, the *E*_CB_ is positioned more negatively than the energy of the H_2_/H^+^ potential, and can split water into H_2_. Similarly, if the *E*_VB_ is more positive than the energy of the O_2_/H_2_O potential, then the water molecule can easily split into O_2_. Similarly, GaN-Ti_2_CO_2_ and AlN-Ti_2_CO_2_ have the ability to produce O_2_ while *E*_CB_ has a positive value and is unable to produce H_2_ due to the narrow bandgap nature. All these findings demonstrate that PN-M_2_CO_2_ (P = Al, Ga; M = Ti, Zr, Hf) vdWHs are the best candidates for photocatalytic water splitting and provide a guideline for designing high performance nanoelectronic, optoelectronic and photocatalytic device applications.

## IV. Conclusions

Using DFT, the calculated electronic band structure and photocatalytic performance of PN (P= Ga, Al) and M_2_CO_2_ (M = Ti, Zr, Hf) monolayers show the potential of such materials in photocatalytic applications. Based on the same hexagonal symmetry and experimentally achievable lattice mismatch of PN (P = Ga, Al) with M_2_CO_2_ (M = Ti, Zr, Hf) monolayers, we have fabricated PN-M_2_CO_2_ vdWHs. The thermal stability of PN-M_2_CO_2_ vdWHs are confirmed by calculating the binding energies, interlayer distance and AIMD, which demonstrate that these materials can easily be fabricated experimentally. All the PN-M_2_CO_2_ vdWHs are indirect bandgap semiconductors, while Type-II[-I] band alignment is obtained for GaN(AlN)-Ti_2_CO_2_[GaN(AlN)-Zr_2_CO_2_ and GaN(AlN)-Hf_2_CO_2_] vdWHs. The PN-Ti_2_CO_2_ (PN-Zr_2_CO_2_) vdWH, PN(Zr_2_CO_2_) monolayer, have greater potential than the Ti_2_CO_2_(PN) monolayer, indicating that charge is transferred from the Ti_2_CO_2_(PN) to PN(Zr_2_CO_2_) monolayer, hence, the potential drop at the interface separates charge carriers (electron and holes). The work function and effective mass of the carriers of PN-M_2_CO_2_ vdWHs are also calculated and presented. For the AlN-Ti_2_CO_2_ effective mass of electrons and holes is greater, and is hence suggested for logic devices, while for the rest of the vdWHs, the effective mass of electrons and holes are lower. Based on *ε*_2_(*ω*) *α*(*ω*), a red (blue) shift is observed in the position of excitonic peaks from AlN to GaN in PN-Ti_2_CO_2_ and PN-Hf_2_CO_2_ (PN-Zr_2_CO_2_) vdWHs, while a significant absorption for photon energies above 2 eV for AlN-Zr_2_CO_2_, GaN-Ti_2_CO_2_ and PN-Hf_2_CO_2_, make them candidates with a good optical profile. The calculated photocatalytic properties demonstrate that PN-M_2_CO_2_ (P = Al, Ga; M = Ti, Zr, Hf) vdWHs are the best candidates for photocatalytic water splitting and provide a guideline for experimentalist to design high performance nanoelectronics, optoelectronic and photocatalytic device applications.

## Conflicts of interest

There is no conflicts to declare.

## Supplementary Material
