# Correction: Revealing the electronic, optical and photocatalytic properties of PN-M_2_CO_2_ (P = Al, Ga; M = Ti, Zr, Hf) heterostructures

**DOI:** 10.1039/d3na90024j

**Published:** 2023-03-06

**Authors:** M. Munawar, M. Idrees, Tahani A. Alrebdi, B. Amin

**Affiliations:** a Department of Physics, Abbottabad University of Science & Technology Abbottabad 22010 Pakistan binukhn@gmail.com; b Department of Physics, College of Science, Princess Nourah Bint Abdulrahman University P.O. Box 84428 Riyadh 11671 Saudi Arabia taalrebdi@pnu.edu.sa

## Abstract

Correction for ‘Revealing the electronic, optical and photocatalytic properties of PN-M_2_CO_2_ (P = Al, Ga; M = Ti, Zr, Hf) heterostructures’ by M. Munawar *et al.*, *Nanoscale Adv.*, 2023, https://doi.org/10.1039/d3na00017f.

The authors regret that the email address of one of the corresponding authors (Tahani A. Alrebdi) was incorrectly listed as taalrebdi@pnu.edu.pk in the original manuscript. The correct email address (taalrebdi@pnu.edu.sa) is listed here.

The Royal Society of Chemistry apologises for these errors and any consequent inconvenience to authors and readers.

## Supplementary Material

